# Study on Auxiliary Rehabilitation System of Hand Function Based on Machine Learning with Visual Sensors

**DOI:** 10.3390/s26030793

**Published:** 2026-01-24

**Authors:** Yuqiu Zhang, Guanjun Bao

**Affiliations:** 1 Department of Biomedical Engineering, College of Design and Engineering, National University of Singapore, Singapore 119077, Singapore; 2 College of Mechanical Engineering, Zhejiang University of Technology, Hangzhou 310014, China; gjbao@zjut.edu.cn

**Keywords:** stroke rehabilitation, deep learning, gesture recognition, gamified design

## Abstract

This study aims to assess hand function recovery in stroke patients during the mid-to-late Brunnstrom stages and to encourage active participation in rehabilitation exercises. To this end, a deep residual network (ResNet) integrated with Focal Loss is employed for gesture recognition, achieving a Macro F1 score of 91.0% and a validation accuracy of 90.9%. Leveraging the millimetre-level precision of Leap Motion 2 hand tracking, a mapping relationship for hand skeletal joint points was established, and a static assessment gesture data set containing 502,401 frames was collected through analysis of the FMA scale. The system implements an immersive augmented reality interaction through the Unity development platform; C# algorithms were designed for real-time motion range quantification. Finally, the paper designs a rehabilitation system framework tailored for home and community environments, including system module workflows, assessment modules, and game logic. Experimental results demonstrate the technical feasibility and high accuracy of the automated system for assessment and rehabilitation training. The system is designed to support stroke patients in home and community settings, with the potential to enhance rehabilitation motivation, interactivity, and self-efficacy. This work presents an integrated research framework encompassing hand modelling and deep learning-based recognition. It offers the possibility of feasible and economical solutions for stroke survivors, laying the foundation for future clinical applications.

## 1. Introduction

Incidence of neurological disorders, represented by stroke, has been on the rise due to the ageing population and the increasing prevalence of unhealthy lifestyle factors such as poor diet, lack of physical activity, and irregular work and rest. According to the World Health Organization [1], about 15 million people worldwide suffer from stroke each year, of which about 6 million die from stroke. According to statistics, the number of stroke patients in China has reached 13 million, and about 2.4 million stroke patients and 1.1 million stroke-related deaths will be added every year [2], making stroke the first cause of adult death and disability in China. The current trend of increasing incidence of stroke is not only a serious threat to the health and quality of life of individual patients but also leads to overloading of the resources of the public health care system and poses serious burdens and challenges to the socio-economic development of the society [3].

Stroke, medically referred to as cerebrovascular accident (CVA), is an acute neurological condition resulting from a sudden interruption of blood supply to the brain. According to the Chinese Guidelines for the Prevention and Treatment of Stroke (2021 edition), it is the leading single cause of disability in clinical practice and is associated with multifactorial structural abnormalities of the cerebral vasculature. These abnormalities ultimately result in central nervous system functional deficits [4]. Studies have shown that in the later stages of the disease, about 80% of the present cases with motor impairment due to damage to the central motor conduction pathway, including motor dysfunction, language deficits, cognitive decline, and emotional problems [5]. Common symptoms of motor dysfunction include unilateral limb weakness, hemiparesis, unsteady gait, and muscle weakness and spasticity [3,5,6], and among them, the hands, as the extremities of the upper limbs, are the most difficult part of the body to recover from stroke, and they are crucial for performing fine motor tasks such as grasping, writing, and eating. Loss of fine motor function in the hands often leads to problems such as loss of grip strength, lack of finger dexterity, abnormal muscle tone, spasticity, and loss of sensation. These problems prevent patients from achieving basic activities such as gripping, dressing, and eating, severely limiting and significantly affecting their ability to participate in daily activities and live independently [6,7]. Therefore, effective assessment and rehabilitation of hand motor function has become a priority issue in the field of rehabilitation medicine [7,8].

Through repetitive exercises in rehabilitation, stroke patients can use neuroplasticity to accelerate the recovery of motor function [7]. Neuroplasticity refers to the brain’s ability to reorganize itself by forming new neural connections in the face of learning or injury. Through sustained, repetitive rehabilitative exercise practice, patients can stimulate the brain’s ability to rewire and compensate for lost function, gradually strengthening and refining the neural connections in the brain related to motor control, and facilitating the activation of neural circuits that may have been affected by the stroke [9].

A pioneering experimental study using non-human primate models conducted by Nudo’s team in the 1990s demonstrated that systematic activation of the motor pathways of the affected limb facilitated adaptive cortical reorganization [10]. This provides theoretical support for a modern three-stage rehabilitation framework: acute clinical treatment, device-assisted recovery training, and home-based self-rehabilitation. However, limited by the allocation of medical resources, about 90% of patients need to be transferred to the home rehabilitation stage after stabilization [11], and the discontinuity of out-of-hospital continuity of care has led to a number of problems: patients generally have a cognitive blind spot in rehabilitation, and lack of systematic knowledge of the science and continuity of the home training programme; the traditional repetitive training model makes it difficult to maintain motivation to participate; coupled with the lack of medical supervision and the increased economic burden, this ultimately results in a significant decrease in patient training compliance three months after discharge [12].

Therefore, effective motor rehabilitation assessment in home and community settings is essential for improving the life quality of stroke patients, not only to encourage the brain to use adaptive strategies to further enhance neuroplasticity through rehabilitation training, which is conducive to the long-term recovery of motor function, but also to real-time effective assessment, which can help the patients to know and understand their own rehabilitation status, and can also significantly enhance the patients’ interest and initiative in rehabilitation. Moreover, real-time assessment can help patients’ awareness and understanding of their own rehabilitation status, and significantly increase their willingness and initiative, which is conducive to the long-term implementation of rehabilitation training to make full use of neuroplasticity to achieve better rehabilitation results.

## 2. Literature Review

With the rapid advancement of technology, artificial intelligence (AI) and deep learning have made significant contributions to the field of rehabilitation medicine, especially in the assessment and recovery of hand motor function in stroke patients [13]. Currently, research on hand rehabilitation for stroke patients focuses on motor assessment and functional recovery techniques [14,15]. These techniques usually involve the use of motion sensors, electromyography, or infrared optical tracking systems to capture detailed motor data [14]. After analyzing the acquired data, physicians can assess movement patterns [5] and detect abnormalities through advanced computer algorithms and track the patient’s recovery progress online over time.

### 2.1. Two Approaches to Functional Hand Rehabilitation

Current research in hand rehabilitation can be broadly categorized into two main approaches: rehabilitation methods based on gesture recognition and computer vision technology and robots for hand function rehabilitation based on wearable devices (Table 1).

#### 2.1.1. Rehabilitation Based on Gesture Recognition and Computer Vision Techniques

This approach favours patient-initiated training, with the system and device playing only an auxiliary role in detection and assessment, and is more suitable for subacute stroke patients. This method uses external devices such as Leap Motion or Kinect V1 and V2 cameras to capture gesture image data, and then uses computer vision or deep learning algorithms to process, recognize, and classify the data; its key advantage is the non-invasive recognition of the input device, which does not require the patient to wear additional equipment and helps to reduce the fatigue of the user, and it can be applied to fine motor rehabilitation and hand function assessment tasks [5,6]. Recent studies have significantly advanced this field by introducing complex architectures for gesture interpretation. For instance, Guo et al. [16] utilized Hierarchical LSTMs for sign language translation, while Jiang et al. [17] and Sincan et al. [18] demonstrated the efficacy of skeleton-aware multi-modal frameworks in improving recognition accuracy. However, the disadvantages are that they require a controlled external environment, they are susceptible to external environmental influences (e.g., lighting conditions and background clutter), and their recognition speed and accuracy tends to be relatively low compared to the second category of methods.

Wang, Z. R. et al. [7] developed a Leap Motion-based VR protocol to target upper limb recovery in subacute stroke patients. By engaging patients in task-oriented scenarios—such as piano-playing and petal-picking—the system aimed to enhance fine motor capabilities, including pinching and finger individuation. Quantitative assessments indicated that this approach significantly reduced task performance time and improved motor function scores (WMFT) relative to the control group. Crucially, the study also provided neuroimaging evidence that such training facilitated adaptive cortical reorganization, specifically increasing activation in the contralateral sensorimotor cortex.

Chien Hoang’s [15] study describes a system that utilizes VR and Kinect technology to promote upper limb motor function rehabilitation in stroke patients. The study tracked upper and lower limb movements in real time through a static human posture grading system and provided an interactive taijiquan exercise programme designed to improve motor control and strength.

#### 2.1.2. Wearable Device-Based Hand Function Rehabilitation Robots

This approach favours passive training for the patient, where the system and equipment are strapped to the patient and bound to the patient’s fingers for rehabilitation, and it is more suitable for early rehabilitation of stroke patients. This approach involves the use of physiological signal sensors such as electroencephalogram (EEG), electrooculogram (EoG) [14], or surface electromyography (sEMG) [19] to capture signals that determine the motor intent of stroke patients. Combined with the data from the Inertial Measurement Unit (IMU) and pressure sensors, these signals are used to relay movement commands to a hand rehabilitation robot or exoskeleton device [20,21,22]. Wearable devices have the advantage of providing more accurate input data and are less susceptible to external interference. However, these devices are usually expensive and need to be calibrated before every use [5]; furthermore, wearing such a device can interfere with the therapist’s movements and decision-making during therapy, and prolonged use may lead to patients’ fatigue.

Cordella et al. [23] developed a camera-based calibration system for the bending sensors of a commercial exoskeleton hand rehabilitation glove, the Gloreha Sininfonia. The system uses eight photocameras to track and reconstruct the angles of 18 reflective markers on the glove, which are integrated with the voltage of the bending sensor to improve the calibration of the exoskeleton angles. Additionally, the system allows the assessment of the hand function by measuring the motion range before and after the treatment, allowing for the assessment of the patient’s progress.

Cisnal et al. [24] developed a hand exoskeleton designed for stroke rehabilitation through RobHand, integrating embedded controls driven by real-time EMG signals, aiming to improve the rehabilitation efficiency through precise real-time control based on EMG signals, and to provide more accurate motor assistance for stroke patients.

### 2.2. Methods of Hand Function Assessments

In terms of assessment, the available assessment methods can be divided into traditional clinical scale assessment and multi-modal quantitative assessment. Commonly used traditional assessment methods include the Fugl–Meyer Assessment for Motor Function (FMA), Action Research Arm Test (ARAT), Brunnstrom Recovery Stages for Hand Function (BRS-H), and Manual Muscle Testing (MMT). In 1966, a Swedish physiotherapist, Brunnstrom, divided the rehabilitation cycle of stroke patients into six stages, with the characteristics of limb and hand movements in each stage as shown in Table 2 [25]. Hand movement characteristics are shown in Table 2. These traditional assessment methods can be achieved through a number of subdivided tests of hand motor function with a final aggregation of the results from a focused perspective such as assessing the comprehensive motor and sensory recovery assessment (FMA), the task orientated assessment of hand function (ARAT), the phased recovery of hand function (BRS-H), and the assessment of muscular strength and mobility (MMT), respectively. However, any scale involves multiple specific sub-tests that require the presence of a trained assessor, which not only risks the limitation of relying on the assessor’s subjective judgement of experience, but also has a significant impact on the patient’s patience for the significant time consuming process of completing all assessment tests, motivation, and confidence in undergoing the recovery assessment [26]. In addition, the lack of quantitative criteria and real-time dynamic feedback of the traditional scale assessment also limits the possibility of linking it with rehabilitation training.

Existing research on quantitative assessment of hand motor function after stroke is gradually expanding from traditional clinical scales to multi-modal, high-precision technology integration. Current research focuses on integrating wearable sensors, kinematic analysis, and machine learning algorithms to improve the objectivity and sensitivity of the assessment. For example, Chenguang Li et al.’s study implemented a multi-modal fusion quantitative assessment framework through kinematic feature extraction based on graph convolutional networks and surface EMG signal processing based on multi-layer long- and short-term memory networks. The framework was implemented by WISEGLOVE19 data gloves containing fibre optic sensors and Thalmic Myo armband containing sEMG sensors to capture real-time kinematic parameters and data inputs, and signal processing and scoring were implemented using graph convolutional networks and multi-layer long- and short-term memory networks, and the results were significantly correlated with year-on-year results of BRS-H scales and FMA scales [27]. In addition, a study by Ming Jack Choo et al. implemented a lightweight wearable hand motion analysis device using an IMU and force sensing resistors, enabling the determination of quantitative data on factors affecting the range of finger motion and fingertip force generation [28]. However, the existing multi-modal quantitative assessment studies still face problems such as the lack of standardized protocols and high costs; and the existing studies only focus on quantitative assessment and do not realize the organic combination of multi-modal quantitative assessment and motor rehabilitation training, so as to form a complete systematic programme of personalized assessment and assisted rehabilitation treatment.

### 2.3. Significance of the Study

In summary, although existing studies of rehabilitation training have achieved high accuracy in controlled laboratory environments, problems and challenges remain in terms of real-time response and environmental adaptability [29]. The operational complexity of existing sophisticated equipment may also lead to difficulties for users, especially for older stroke patients who may not be familiar with advanced technologies [30]. In addition, most stroke assessment and rehabilitation treatments are currently only available in hospital facilities and laboratories, which limits accessibility to patients requiring ongoing care and practice, and the transition from clinical rehabilitation to home- or community-based treatment remains a gap in the field [6,7], with a supportive system framework of portable and family-friendly rehabilitation equipment adapted to the home or community setting not yet fully established [31]. Therefore, in order to bridge the gap between hospital-based and home-based therapeutic rehabilitation care, scalable, adaptable, and cost-effective hand movement assessment and rehabilitation solutions for stroke for home and community settings will be a key area for future research and development.

## 3. Tracking Principle of Leap Motion Controller 2 Visual Sensor

**Figure 1:**
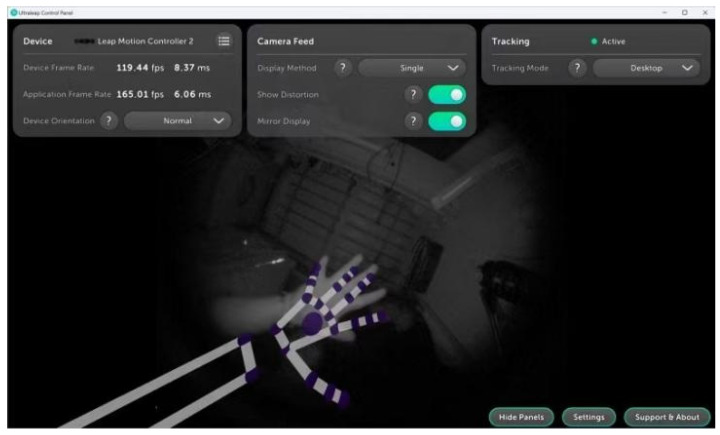
Example of real-time hand skeletal point localization tracking shown after processing by the Ultraleap driver.

The Leap Motion Controller 2 sensor module reconstructs the motion information of the palm of the hand in real-world 3D space based on the footage captured by the two built-in monochrome infrared cameras (resolution 640 × 480) from different angles with the driver provided by the official website of Ultraleap [31], achieving sub-millimetre high-precision and resolution tracking (Figure 1), which can capture the subtle movements of 22 bone nodes of each hand in real time. A structured light field is projected by an active 850 nm infrared LED array to enhance the robustness of feature point recognition, the built-in driver corrects the original image distortion based on distorted mapping of a 64 × 64 mesh with bilinear interpolation, binds the hand bone modelling and joint features, and generates discrete tracking frames and introduces a frame interpolation algorithm to optimize the temporal continuity to reduce rendering delay and motion jitter.

By accessing the Leap Motion SDK and Leap API integrated in Unity, combined with the drivers provided by the official Ultraleap website, it is possible to display the real-time virtual skeletal model after visualization in the Unity development platform [32]. The virtual skeleton of the hand also has its corresponding anatomical bone structure model [31].

By accessing the Unity platform, the Ultraleap tracking system provides accurate physical tracking data of the hand, including spatial position, orientation, and rotation angle information of each joint point within the virtual space, and spatial position information of the three additional joint points at the palm, wrist, and elbow of each hand. As shown in Figure 1, the Ultraleap hand skeleton model has four bones per finger (metacarpal, proximal phalanx, middle phalanx, and distal phalanx) [31] due to the thumb’s lack of an intermediate phalanx in the real anatomy, the same effect is achieved in the hand tracking model by compensating for the same effect by making the thumb metacarpal bone length to be zero.

## 4. Model and Optimization of the Deep Residual Network for Stroke Gesture Recognition

### 4.1. Current Issues and Challenges

**Figure 2:**
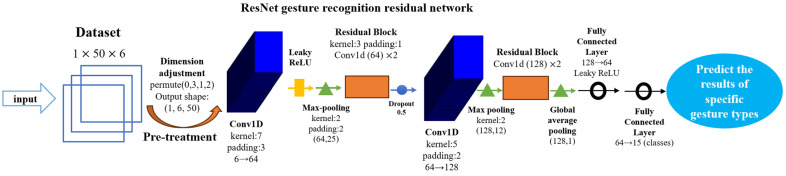
Schematic of the ResNet gesture recognition deep residual network architecture model.

In recent years, deep learning algorithms have provided unprecedented accuracy and real-time processing capabilities in the field of hand movement recognition and movement intent prediction. Key architectures such as convolutional neural networks (CNNs) and residual block structures have been widely used for hand movement recognition, offering unique advantages. Deep residual networks (ResNet), an important branch of deep learning with significant hierarchical features, have been very effective in processing visual data, which makes it particularly suitable for gesture recognition and tracking [33]. While emerging architectures such as transformers and graph neural networks (GNNs) show promise in feature representation, this study prioritizes the ResNet architecture for its proven balance between high accuracy and computational efficiency, ensuring stable real-time performance on consumer-grade hardware for home rehabilitation. The ResNet deep residual network model for gesture recognition used in this study mainly contains components such as an input layer, a feature extraction layer (convolutional layer, residual module, pooling layer), and a deep neural network classifier, and the overall structure of the model is shown schematically in Figure 2.

#### 4.1.1. Input Layer and Data Preprocessing

The input layer accepts normalized per-frame hand skeletal joint coordinate data and their corresponding gesture labels. Each input sample contains 3D spatial coordinate information (x1, y1, z1, x2, y2, z2) of the two endpoints of 50 skeletal line segments consisting of 22 joints of each hand, forming an input tensor of dimension 1 × 50 × 6. The original data is shaped as (1,1,300), and the dimensional transformations are implemented sequentially through three functions: view, permute, and reshape to finally form the input tensor with the correct dimension (1,6,50), which transforms the joint coordinate data into a one-dimensional convolutional processable sequence, and each coordinate component is used as an independent channel to be processed through the sliding window mechanism (window size = 1, stride = 1) to achieve feature extraction.

The data preprocessing process consists of normalization and data enhancement strategies.

##### Standardization

Z-score standardization was used to calculate and save the mean and standard deviation of each coordinate dimension:

(1) $$\;X_{norm}=\frac{X\;-\;\mu }{\sigma \;+\;\varepsilon }$$

where μ denotes the characteristic mean, σ is the standard deviation, $\varepsilon =1{\;\times \;10}^{-8}$ is the numerical stability constant, and μ is the final obtained mean. The Z-score standardization process unifies the data of different magnitudes into the same magnitude, effectively eliminating the magnitude differences in the amplitude of the motions of different joints in the dataset.

##### Data Enhancement

Four types of random data enhancement strategies are set up, and the trigger probability is set separately to enhance the anti-interference ability of the model. The four types of data enhancement are joint flipping process, random scaling process, Gaussian noise injection process, and random joint masking process, and the enhancement operations are all performed at the CPU when the data are loaded.

The joint flipping process simulates the symmetric left and right movements of the human body (e.g., left and right hand movements are interchanged) through the flipping tensor to enhance the understanding of the spatial symmetry in the model, and the mathematical expression is shown as below:

(2) $$X^{\prime }\left[w,j,c\right]=X\left[w,J-j-1,c\right]\;\;\;\;\;\;\;\;\forall j\in \left[0,J\right),c\in \left[0,C\right)$$

Here the input data tensor is $X\in R^{w\times J\times C},\;w=window\;size=1,\;J=num\;joints=50,\;and\;C=num\;coords=6$. The independent triggering probability of joint flipping is 50%.

The random scaling process scales the data (0.8 to 1.2 times) by uniform sampling to simulate the difference in hand size of users with different body sizes and to enhance the model’s immunity to scale variations, with an independent trigger probability of 50%, as shown in the mathematical expression as below:

(3) $$X^{\prime }=X\cdot s,\;\;\;\;\;\;\;s\sim U(0.8,\;1.2)$$

Gaussian noise injection is handled by adding Gaussian noise to simulate sensor acquisition errors to prevent the model from overfitting to the input data, which is further adjusted by the base noise intensity (σ=noise std=0.05) and the dynamic attenuation factor (r∈[0, 1]) with an independent trigger probability of 40%:

(4) $$X^{\prime }=X+\epsilon \cdot \sigma \cdot r,\;\;\;\;\;\;\;\;\;\epsilon \sim N\left(0,1\right)$$

The random joint masking process randomly selects eight joints among fifty joints to mask their six coordinate values (x1, y1, z1, x2, y2, z2) to simulate partial sensor failure scenarios and force the model to learn redundant feature representations. The treatment is independently triggered with a probability of 30% and is mathematically expressed as follows (*M* is the set of randomly selected joints):

(5) $$X^{\prime }\left[w,j,c\right]=\left\{\begin{array}{c} \;0,\;j\in M\;\\ X\left[w,j,c\right] \end{array}\right.$$

#### 4.1.2. Feature Extraction Layer

The feature extraction layer contains a convolutional layer, a residual module, and a pooling layer, the structure of which is shown as below:

(a) Convolutional layer:

The convolutional layer is the core component in a convolutional neural network, which is mainly used to automatically extract local features (e.g., the edges and textures of an image, etc.) from the input data. The core idea is to generate a new feature map by sliding a convolutional kernel (filter) over the input data and performing local computations. The one-dimensional convolutional layer (Conv1D), as the core operation in deep learning for processing one-dimensional sequential data (e.g., time series, text, audio signals), slides the convolution kernel in a single direction (length), and the mathematical expression is shown in Equation (6), which is suitable for dealing with the one-dimensional joints and spatial coordinate sequences involved in this work:

(6) $$\;(X*W)\left[i\right]=\sum_{k=0}^{K-1}X[i+k]\cdot W[k]$$

Here *X* is the input sequence (length *L*), W is the convolution kernel (length *K*), and * is the convolution operation.

The convolutional layer of this study model uses two independent Conv1D convolutional layers, the first Conv1D convolutional kernel W of length *K* = 7, and maintains the input length by symmetric padding (padding size = 3), the number of channels is multiplied from 6 to 64 times, and then after a Leaky ReLU activation function, pooling layer, residual module, and after a Dropout regularization layer (its mathematical expression shown in Equation (7), with Dropout probability p=0.5) followed by a second independent Conv1D convolutional layer; the second Conv1D layer uses a convolution kernel W of length *K* = 5, and maintains the input length by padding = 2, and the number of channels is multiplied from 64 to 128 times, gradually extracting deep features and capturing high dimensional information:

(7) $$\;h^{\left(l+1\right)}=Dropout\left(\sigma \left(W^{\left(l\right)}h^{\left(l\right)}+b^{\left(l\right)}\right)\right),$$

here *σ* is the activation function.

(b) Residual module:

In deep convolutional neural network architecture, the residual block structure effectively mitigates the gradient decay problem by introducing a constant mapping mechanism [34]. The residual block used in this model is designed with a two-branch structure: the main path contains two cascaded Conv1D convolutional layers (kernel size = 3, padding = 1), each of which is followed by a batch normalization process with a Leaky ReLU nonlinear activation function  (a=0.01). The bypass path maintains the original input dimensions and finally achieves feature fusion by tensor summation, and finally integrates the Dropout layer to prevent overfitting. At the network architecture level, residual modules are strategically deployed at key locations in the feature extractor: the first residual module is inserted after the 64-channel convolutional layer to enhance shallow feature reuse, and the second residual module is embedded after the number of channels is scaled up to 128 to enhance deep feature representation.

(c) Pooling layer:

The pooling layer is a type of downsampling that ensures feature invariance and achieves feature dimensionality reduction, and to some extent also controls overfitting. The model in this study achieves feature downsampling after two Conv1Ds by a maximum pooling operation (kernel size = 2), the maximum value in the region is retained as the pooled value in that region; and this work uses adaptive pooling after the second residual module, which adapts to variable-length sequences by making the inputs of different lengths uniformly dimensional.

#### 4.1.3. Deep Neural Network Classifier

In terms of deep neural network classifier design, this study adopts a two-stage fully connected layer architecture to achieve feature dimensionality reduction and category probability mapping, which consists of two fully connected layers to form a hierarchical feature compression structure: the first layer linearly projects the 128-dimensional spatial features to the 64-dimensional dimension, and introduces a nonlinear transformation through the Leaky ReLU activation function (a=0.01); to prevent overfitting, a Dropout regularization layer with a dropout probability of p=0.5 is applied to the fully connected layer; the sub-layer further maps the 64-dimensional spatial features to the target category dimensions, and the final output is the binary classification results for each 15 gesture types in this work.

### 4.2. Model Training and Optimization Strategies

#### 4.2.1. Application of Focal Loss for Class Imbalance

The model in this study uses the Focal Loss function to address the category imbalance problem by adjusting the weight of the difficult and easy samples so that it reduces the loss of the samples allocated to the well categorized samples. The formula is displayed as shown below [35]:

(8) $$FL\left(p_{t}\right)\;=\;-{\alpha_{t}\left(1-p_{t}\right)}^{\gamma }log\left(p_{t}\right)\;$$

where the focusing parameter (γ=1.5) increases the sample weight and the category weight parameter $\alpha_{t}=\frac{1}{\ln\left(N_{t}+1.5\right)}$. The ${\left(1-p_{t}\right)}^{\gamma }$ term automatically reduces the weight of easy-to-categorize samples, and the weight tends to be zero when $p_{t}\to 1$. The $\alpha_{t}$ parameter is weighted according to the category frequency to deal with the category imbalance problem, and the model pays more attention to the difficult-to-categorize samples and a few categories through the double adjustment mechanism.

#### 4.2.2. Mixed Precision Training

Forward propagation: matrix operations using FP16 precision are mathematically expressed as follows:

(9) $${\hat{x}}_{fp16}\;=\;-Conv1D\;\left(x_{fp16};W_{fp16}\right)$$

Loss scaling: the loss value is scaled up by S times to prevent gradient underflow, which is mathematically expressed as follows:

(10) $$\;L_{scaled}\;=\;L\times S\;\;\;\;\;\;\;\;\;\left(S=2^{16}\right)$$

Parameter updated: The scaled gradient is converted back to FP32 for parameter updated, which is mathematically expressed as follows:

(11) $$W_{fp32}=W_{fp32}-\eta \cdot \left(\frac{\nabla W_{fp32}}{S}\right)$$

The above optimization strategy can reduce the memory footprint by about 50% and increase the training speed by 1.5 to 3 times while maintaining the model accuracy at FP32 precision level.

#### 4.2.3. Dynamic Learning Rate Scheduling

Using the dynamic learning rate scheduling (ReduceLROnPlateau function) strategy, the logic is to compute the validation set F1 score after each epoch, and when the F1 score does not improve by more than 0.001 for five consecutive epochs, the learning rate is decayed to 50% of the current value and the minimum learning rate is restricted to be $1\times 10^{-5}$, which is mathematically expressed as follows:

(12) $$\;lr_{new}=\max\left(lr_{current}\times 50\%,{lr}_{min}\right)$$

#### 4.2.4. Early Stopping Mechanism

The early stopping mechanism prevents the model from continuing to overfit when the performance of the validation set stagnates, which saves about 20–30% of the training time on average and retains the best model parameters to improve the generalization ability; the minimum improvement threshold Δ = 0.001 and the maximum tolerated epoch number patience is 15.

#### 4.2.5. Assessment Indicators

Due to the uniform distribution and consistent weight share of each gesture category, this study adopts the average F1 score (macro-averaged F1 score) as the core model evaluation metric, and the validation accuracy (Val Acc) as the auxiliary metric, whose mathematical expression is shown as below:

(13) $$\;\;\;\;\;\;\;\;\;\;\;\;\;\;\;\begin{array}{c} \;Macro\;F1=\frac{1}{C}\sum_{c=1}^{C}{F1}_{c},\;\\ \;F1=2\cdot \frac{Precision\cdot Recall}{Precision+Recall}=\frac{2\;TP}{2\;TP+FP+FN} \end{array}$$

(14) $$Val\;Acc=\frac{TP}{TP+TN+FP+FN}$$

Here *TP* is the true case, i.e., the sample that the model correctly predicts is a positive class; *TN* is the true negative case, the sample that the model correctly predicts is a negative class; *FP* is the false positive case, where the model incorrectly predicts a negative class is a positive class; and *FN* is the false negative case, where the model incorrectly predicts a positive class is a negative class.

## 5. Data Set Construction and Experimental Analysis

### 5.1. Figures, Tables, and Schemes

Leap Motion sensors are widely used to capture highly accurate hand skeletal joint localization and spatial coordinates in real-time. After reviewing a large number of studies and considering various factors, this study chooses to obtain the source data of spatial coordinates of hand skeletal joint points in the virtual space displayed by the real-time Leap Motion Controller 2 with reference to a specific gesture of the FMA scale via the Unity platform, and using the open-source Leap Motion Dynamic Hand Gesture (LMDHG) database [36] for the hand skeletal joint point mapping as the mathematical modelling of the hand skeleton for training ResNet.

#### 5.1.1. LMDHG Data Set

**Figure 3:**
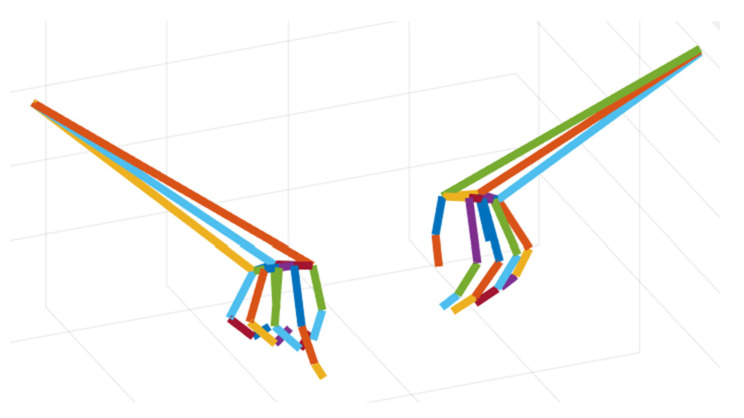
Skeletal model distributions for the LMDHG dataset plotted by MATLAB. Different colors denote different hand joints (or skeletal segments).

The LMDHG data set is an open-source collection designed for dynamic gesture recognition, unlike existing datasets, this provides sequences of unsegmented gestures performed with either one hand or both hands captured by 21 individual participants amounting to 608 gesture instances by using the Leap Motion sensor [36]. After reading the mat files in the LMDHG data set through the MATLAB (R2024a, MathWorks) program, the number of frames, serial numbers, category labels, and corresponding 3D coordinate information for each instance in the LMDHG data set can be obtained after dumping the csv files (as shown in Figure 3).

#### 5.1.2. Mathematical Modelling of the Hand Skeleton and Data Set Composition

**Figure 4:**
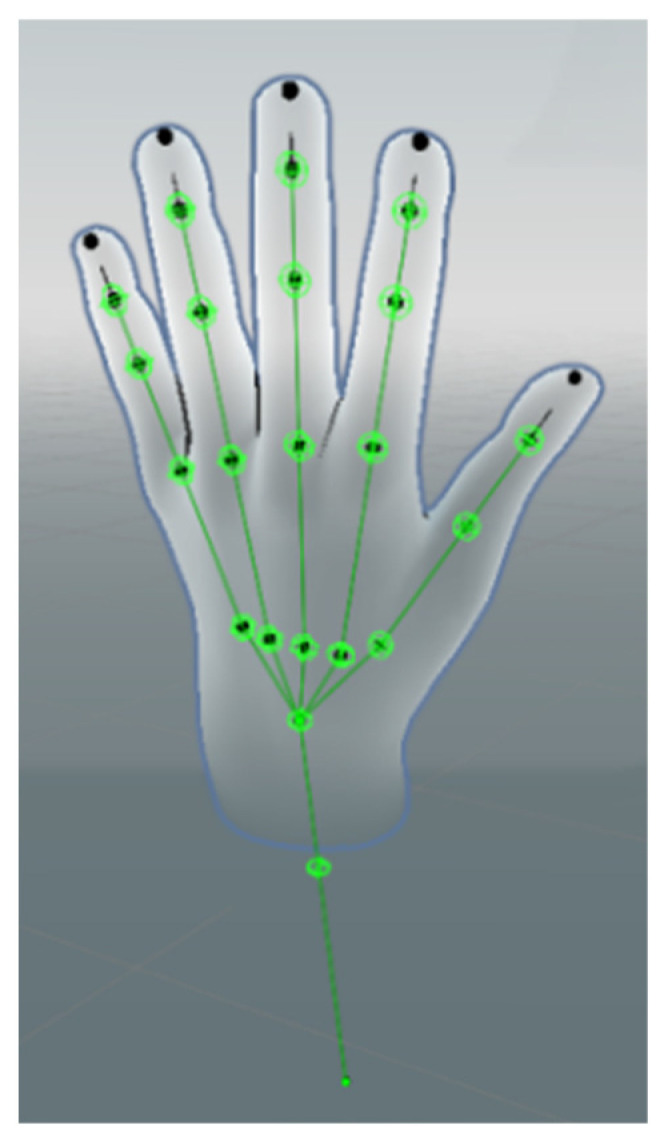
Skeletal model joint points for Leap Motion Controller 2 (single hand). The black markers at the fingertips denote visualization points representing the distal ends of the fingers and are not included as skeletal joint points in the model input.

Due to the upgrade of the Ultraleap device (Leap Motion Controller 2, Ultraleap Ltd., Bristol, UK) and its driver, the way the LMDHG skeletal model line segments composed collection in the year of 2017 is partially different from the way the original input skeletal model line segments (22 joint points on each hand) composes now as shown in Figure 4. By parsing the initialized connectivity map (mapping between joints) of the LMDHG, a total of 50 line segments (sequences) were constituted with mapping relationships to the skeletal joint points of the hand as shown in Table 3.

The above skeletal model construction and joint point mapping strictly follow the hand anatomy: wrist → palm → proximal → intermediate → distal biomechanical hierarchy, with biomechanical accuracy, and enhance the stability of the palm region through redundant connections (e.g., line sequence numbers 10, 14, 18, 22, etc., in Table 3) and simplify the logic of left- and right-handed data processing through symmetrical design.

Referring to Table 3, the gestures used in the gesture evaluation module and the subsequent rehabilitation training with reference to the FMA scale (as shown in Table 4) are not compatible with the existing dynamic gesture types (drawing C-shape, scissor gesture, finger sliding, etc.) in the LMDHG. Therefore, this work mimics the mapping of hand bone joints in the LMDHG data set as the mathematical modelling of hand bones for training, and record 15 static gestures (the first 7 gestures are modified according to the Leap Motion Controller 2’s features and the last 8 gestures are static gestures referenced to everyday use, which are ultimately used in the Static Gesture Recognition Rehabilitation Game Module) that comply with the FMA scale using the C# algorithm mounted on the Unity platform via the Ultraleap driver. Although the training dataset is composed of static frames for classification, in the subsequent Unity integrated system, the model is applied to a continuous sensor data stream (120 Hz) over a duration through a time component to achieve dynamic monitoring of the gesture execution process. Specifically, the content of the data set is the spatial coordinates of the hand skeleton joint source data in the virtual space over a number of frames labelled with the category of static gestures at the time of the recording. A total of 19 healthy right-handed participants (8 males and 11 females, aged 22 to 35 years, with an average age of 28.2 ± 3.5 years) were recruited for this study. All participants had no history of upper limb motor dysfunction or neurological diseases. Data collection was carried out in a quiet laboratory environment. Participants placed their hands above the sensor detection area of Leap Motion Controller 2 in a natural sitting position. The detailed composition of the data set can be seen in Table 5, with a total data volume of 502,401 frames, corresponding to samples extracted from multiple repeated recordings of 19 participants. All cross-validation experiments in this study are conducted at the frame level. Accordingly, the reported performance reflects frame-wise discrimination and does not directly imply subject-level generalization.

The dataset shown in Table 5 was divided into training and validation sets using a Stratified K-Fold cross-validation (k = 5) at the frame level, with an 8:2 ratio. While subject-independent splitting is often preferred for behavioural analysis, this study focuses on recognizing standardized static physiological structures (FMA gestures) where anatomical consistency outweighs behavioural idiosyncrasies. To mitigate potential overfitting to specific subjects and ensure the model learns robust geometric representations rather than memorizing individual hand shapes, we implemented rigorous data augmentation strategies (detailed in Section 4.1.1), including random scaling (simulating different hand sizes) and Gaussian noise injection (simulating sensor variances). These measures effectively expand the diversity of the feature space, allowing the frame-level evaluation to reliably reflect the model’s capability in recognizing standard rehabilitation postures. The data set was also trained applying real-time enhancement (flipping, scaling, noise, joint occlusion) and category-balanced sampling to the training sets.

### 5.2. Experimental Analysis

In this section, this study conducts experimental analysis based on the optimized ResNet model and the spatial coordinates data set of the hand bone joint points constructed above. The corresponding experimental environment and experimental parameters are selected, and the improved ResNet model is used for gesture recognition in combination with the evaluation indexes (Macro F1 and Val Acc) for model performance evaluation in deep learning, and the experimental data are analyzed in the ablation experiments.

#### 5.2.1. Experimental Environment and Parameters

The deep learning experiments were conducted on a Windows 11 system equipped with an Intel(R) Core(TM) i9-14900HX CPU, an NVIDIA RTX4060 GPU, and 32 GB of RAM. The software environment utilized PyTorch 2.0.1, Python 3.9.16, and CUDA 11.8.

The specific experimental parameters are set as follows: batch size is set to 32, workers are set to 4, the AdamW optimization strategy is adopted, the initial learning rate is 0.001, and the network model is set to a total of 200 epochs with an early stopping patience of 15. Input Size (Pre-treated) is (1,50,6).

#### 5.2.2. Results and Analyses of Ablation Experiments

**Figure 5:**
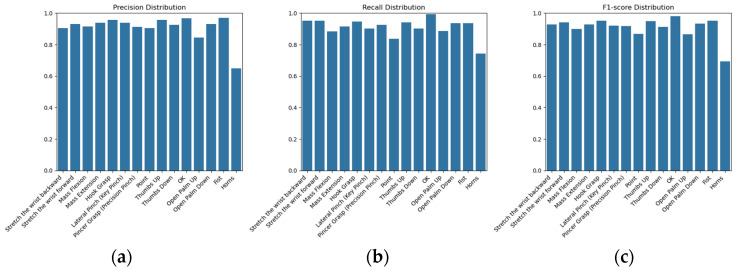
Classification performance metrics for the 15 static gestures, including (**a**) precision, (**b**) recall, and (**c**) F1 score distributions.

In order to systematically evaluate the performance of the selected model architecture, this study designs four sets of ablation comparison experiment (Table 6), covering the independent and combined optimization of the underlying MLP, CNN, residual linkage, LSTM, and Focal Loss, with scenario NO. 5 being the final ResNet model in this study. The following specific analyses are developed in conjunction with the results of the experimental data in Table 6 and Figure 5, where the experimental results are the Macro F1 and Val Acc results of the best-performing model obtained after five crossover experiments.

By comparing the results of the ablation experiments, it is evident that the ResNet-based gesture recognition model composed of a conventional CNN backbone and deep residual blocks, optimized with Focal Loss, achieves the best overall performance among all evaluated configurations. As reported in Table 6, this best-performing configuration does not include an LSTM module and attains a Macro F1 score of 91.0% and a validation accuracy (Val Acc) of 90.9%.

The prediction accuracy, recall, and F1 score distributions for each of the 15 static gestures produced by this final ResNet gesture recognition model are shown in Figure 5, based on the best-performing configuration identified in the five-fold cross-validation using the cnn_im2_fold_1_best.onnx model.

## 6. Hand Function Assessment and Motor Rehabilitation System for Stroke Patients

**Figure 6:**
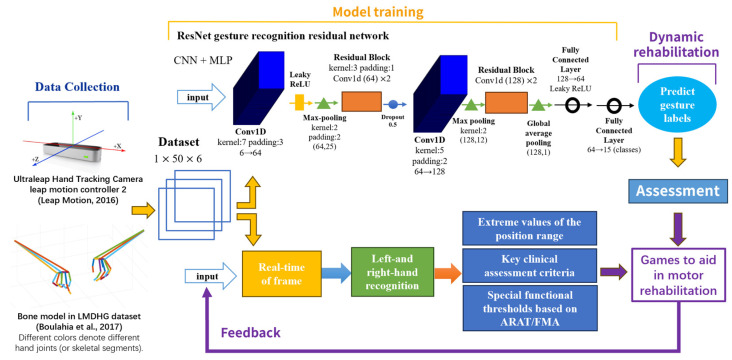
Technology roadmap for the development of a rehabilitation system for hand function assessment in stroke patients [32,36].

The proposed assessment and rehabilitation system integrates three core components in a closed-loop workflow (Figure 6). First, Leap Motion 2 provides non-invasive capture of real-time, high-precision 3D skeletal hand data (22 joint points for each left and right hand, sampled at 120 Hz). Second, a ResNet-based gesture recognition model identifies movement intent through static gesture classification. Third, continuous kinematic tracking is used to analyze the temporal characteristics of gesture execution. This hybrid approach allows the system to assess not only the final hand pose but also the dynamic process of reaching that pose through the C# algorithm development supported by the Unity platform.

This study achieves immersive augmented reality interactive interface through the Unity platform access to Leap Motion 2, respectively, by gesture determination and C# algorithm implementation of the two assessment modules, combined with the game module through the visual and auditory feedback. This design aims to improve the patient’s sense of participation and willingness to train and further enhances the effect of rehabilitation training.

### 6.1. Parameter Design and Definition

Referring to the FMA scale, ARAT scale, and BRS-H scale, the normal joint range of motion values of both hands are shown in Table 7 (the average value of each scale is taken as a reference) and combined with the key clinical assessment criteria of each scale (Table 8), and with the examples of ARAT/FMA-based special function thresholds (Table 9), quantitative data thresholds are formed, thus obtaining real-time hand bone position information to support algorithmic assessment.

### 6.2. Code Design for Quantitative Evaluation of Real-Time Motion Range

The HandAssessor.cs code serves as the core of the real-time quantitative range of motion assessment in this study. The quantitative analysis of motor function parameters was achieved by capturing real-time per-frame biskeletal data inputs through the Leap Motion Controller at a sampling rate of 120 Hz by using the above covariate design and defining the specific data as a paradigm (Table 7, Table 8 and Table 9). The code is intended to be consistent with traditional assessment scales (BRS-H, FMA, ARAT) and achieves algorithmic equivalence with traditional scales through a three-phase logical architecture (data acquisition, feature extraction, clinical mapping).

#### 6.2.1. Multi-Modal Feature Extraction

Joint kinematic parameters: including wrist extension angle (based on the forearm-palm normal vector angle), elbow flexion angle (calculated from the upper arm–forearm vector space relationship), and thumb-to-metacarpal distance (measuring the Euclidean distance between the distal phalanx of the thumb and the fifth metacarpophalangeal joint).

Task completion metrics: an event counter was used to record thumb-to-palm maneuvers per unit time that met anatomical criteria (the contact distance threshold was set at 5 cm, in line with ARAT functional contact norms).

#### 6.2.2. Clinical Scale Mapping Algorithm

(a) FMA scoring model: establishing a weighted integral equation, where the degree of wrist extension (*S_w_*), the degree of elbow flexion (*S_e_*) and thumb-to-palm success rate (*S_t_*) were used as full scoring benchmarks of 70°, 150°, and 100%, respectively.
(b) ARAT scoring model: a progressive scoring strategy is used, and the ARAT penalty mechanism is triggered when wrist extension is less than 56°.
(c) BRS-H staging decision tree: building a four-stage classifier based on wrist kinematics, i.e., stages III–VI.
(d) Dynamic feedback mechanism: continuous updating metrics during a 30 s assessment cycle. Objectively quantified by algorithms, the mechanism avoids errors caused by disparities between raters of traditional scales and enables the analysis of bilateral motor asymmetry through a modular design (independently maintained instances of right- and left-handed assessments).

The decision logic prioritizes clinical validity through threshold-driven condition scores. For example, thumb opposition is labelled as ‘success’ only if the thumb-to-pinky distance is less than 0.05 units (Table 8), reflecting the functional contact criteria of the ARAT. Similarly, the BRS-H staging follows a validated motion range (Table 9) to ensure consistency with rehabilitation benchmarks. Due to the inability of existing input devices to enable grip sensor detection, MMT-related assessment is a systematic shortcoming of existing purely vision-based sensors.

### 6.3. Design and Realization of the Processes of the Modules of the Overall System

Each module uses a finite state machine to realize the assessment module process and the rehabilitation training module process, and the core process includes a voice guidance stage, countdown preparation stage, real-time detection stage, and result feedback stage.

After entering a new scene, a TTSVOX-synthesized voice provides scene prompts and gesture instructions. These audio cues are combined with on-screen text to enhance user comprehension. The voice guidance of each scene is about 10 to 14 s, after the voice guidance is finished, it automatically enters the countdown preparation stage, enters the 10 s countdown, accompanied by the rotation of the start button countdown progress bar display (during the period, you can click the start button at any time to enter the assessment process or the rehabilitation training game process), and automatically enters the assessment process or the rehabilitation training game process after the end of the 10 s countdown. Next, the real-time monitoring phase is performed in Unity. Leap is carried out during the training or evaluation process, listening to the event structure of the remaining components through the Unity event system and constructing real-time visual feedback of completion or non-completion accordingly.

At the end of the real-time monitoring phase, the system will enter the structural feedback phase, displaying the corresponding result text and playing the corresponding result tone synthesized by TTSVOX. If removing the event listener to release the resources, the system will display the continue button and enter the 10 s countdown (accompanied by a rotating countdown bar displaying the continue button, during which you can click the continue button at any time to switch the scene), and at the end of the 10 s countdown, the system will automatically load the next scene using after the 10 s countdown, the next scene will be loaded automatically using Scene Management.

### 6.4. Design and Realization of Evaluation Module

The BRS-H will be used as the primary qualitative scale to assess hand recovery, supplemented by the FMA and ARAT as quantitative references. The aim of this study is to provide assessment and assist rehabilitation for the BRS-H partially separated motor period and the recovery exercise in the future.

**Figure 7:**
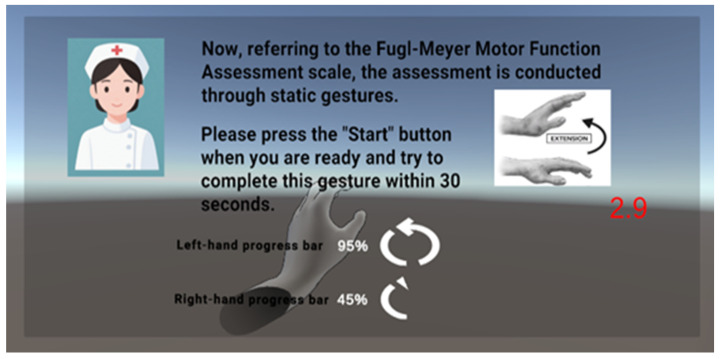
Example scenarios for each gesture (currently showing the first action wrist extension backwards based on FMA gesture evaluation). The red numeric display represents a 30-s countdown timer, with the value decreasing over time after the task starts.

**Figure 8:**
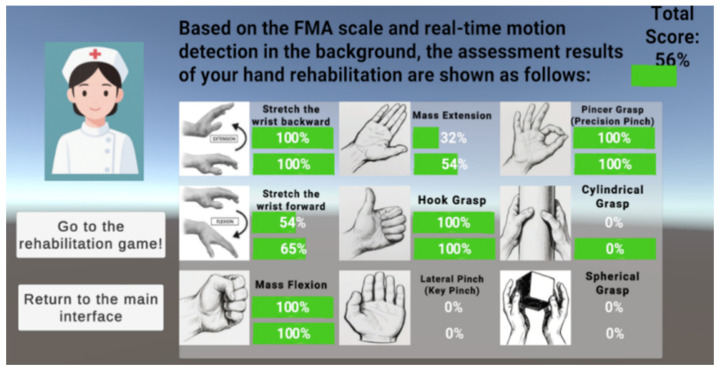
Presentation of the final results of the gesture assessment based on the FMA scale.

The study currently implements the following two main assessment methods:

(1) The first is a reference FMA scale combined with the Leap Motion Controller 2 hand joint point detection range, which initially assesses hand fine motor skills by detecting nine specific FMAs to assess whether a gesture is correctly executed as instructed and can be autonomously maintained for a period of time. The system assesses the dynamic stability of the hand function by monitoring whether the gesture can be correctly executed and autonomously maintained without tremors or deviation for a period of time. Based on Leap Motion, the original hand data input is realized. Here, the first seven gestures are combined with ResNet gesture recognition model and C# algorithm to build a two-channel verification mechanism for rule matching, and the last two gestures (column grip and ball grip) are realized by the Unity engine and C# algorithm for rigid-body interaction determination. The dual-channel verification mechanism is to use Unity’s event system to implement the double-listening verification of the deep learning model mount component to judge the static gesture events, and the C# gesture pose detection component to detect the static gesture events, in order to further improve the accuracy of gesture recognition. Dual-listening verification is to listen to the gesture detection events of the C# gesture pose detection component and the gesture detection events of the ResNet gesture recognition model mounted on the left and right hands, respectively, and if the dual-channel is verified and passed, then the white progress bar and progress value in the virtual space corresponding to the left and right hands will be increased, so as to achieve the augmented reality interaction and real-time visual feedback (as shown in Figure 7). Upon completion of all nine individual FMA gesture detections, the system presents a final visualization of the assessment results, as illustrated in Figure 8.

The C# gesture pose detection component is a Unity prefabricated component for judgement of specific bone construction (limited to a special rotation angle) implemented by C# code, and each finger is divided into joints for judgement detection—the thumb is divided into three joint judgements for the proximal joint point, the middle joint point, and the distal joint point; the remaining four fingers are divided into two joint judgements for the proximal joint point and the middle joint point. There is an internal joint rotation threshold of 30, i.e., the deviation cannot exceed 30 degrees, and the deviation calculation formula is as follows:

(15)  Flex=FlexionCurl=30,   Abd=AbductionSplay=30

where *Flexion* refers to the angle of flexion of the joint towards the palm, *Curl* refers to the degree of flexion of the finger from full extension to full fist clenching, *Abduction* refers to the angle of lateral movement of the finger away from the neighbouring finger (e.g., the index finger is separated from the middle finger), and *Splay* refers to the degree of overall spreading that includes more than one interphalangeal finger.

**Figure 9:**
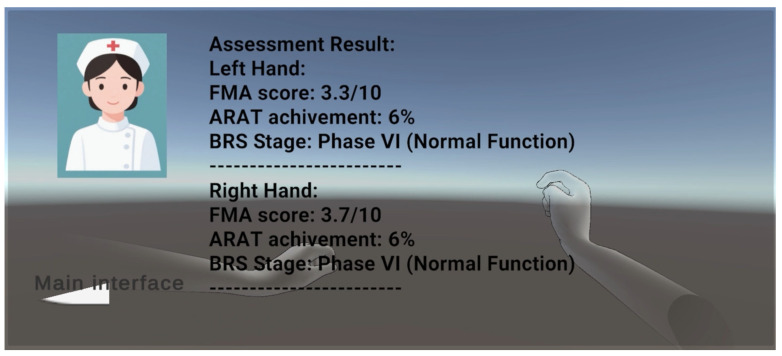
Example of C#-based interface for overall algorithmic evaluation results.

(2) The second method is to use HandAssessment.cs code reference to define the normal reference joint motion range for quantitative assessment of three dimensions (BRS-H, FMA, ARAT), which not only realizes real-time quantitative assessment but also combines it with the gesture determination rehabilitation training game to realize multi-dimensional real-time assessment feedback after the training is completed. The sample interface diagram of the overall algorithm evaluation result based on C# is shown in Figure 9.

### 6.5. Design and Implementation of Game Logic Module

Regarding the game logic design, there are two interactive games for rehabilitation training:

(1) One is an object virtual interaction game by grasping block cubes and placing them into the adsorption box;
(2) The other is a static gesture determination game using the same technical method of gesture evaluation.

Both games can be implemented using the Leap Motion Unity API, and the scene development build and game logic code implementation have been completed and is expected to provide a dynamic and engaging approach to motor rehabilitation and continuous assessment.

**Figure 10:**
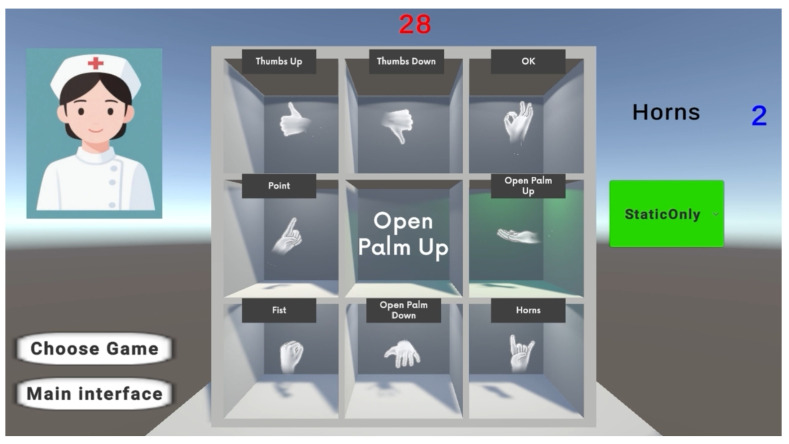
Gesture recognition during rehabilitation games. The red numeric display represents a 30-s countdown timer that decreases over time, while the blue numeric display indicates the cumulative count of correctly detected gestures.

The game logic for gesture recognition is as follows: you can wait for the 10 s countdown after the tone is over, or you can start the game directly. The game starts with a 30 s countdown and a randomly generated static gesture target in the upper right corner. If the static gesture target is completed, the count in the upper right corner will increase by 1, and the light in the corresponding square in the scene will turn green to give a reminder. The display text in the middle of the scene also shows the name of the currently detected static gesture in real time. Meanwhile, at the beginning of the game, an evaluation algorithm is loaded in the background to assess the motion state of the hands. Finally, at the end of the game, a pop-up window in the centre of the scene displays the number of static gestures completed within the time limit and the evaluation results during the game (shown in Figure 10).

During the gesture game, Gesture Recognition Rehabilitation Game Code Implementation, HandAssessor.cs code is mounted in the background. After completing a gesture recognition rehabilitation game, the system can achieve not only to display the completed static gesture target but also to display the three-dimensional (BRS-H, FMA, ARAT) assessment results after the C# algorithm assessment in the rehabilitation game, which may help to enhance the patient’s own rehabilitation situation awareness, potentially improving their willingness to recover and self-confidence.

**Figure 11:**
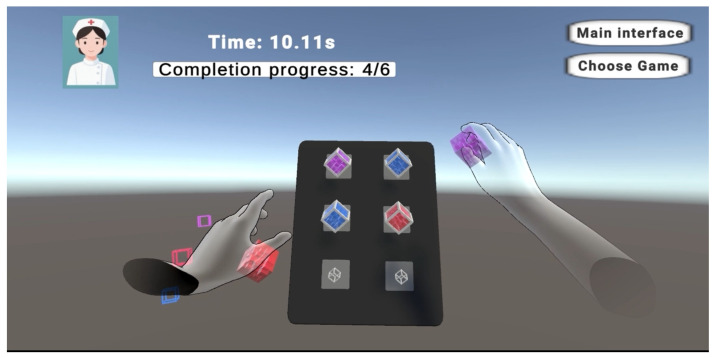
Schematic diagram of object interaction game. Different colors represent three distinct rigid bodies, for which collision detection and positional computations are performed independently.

The game logic for object interaction is as follows: you can wait for the 10 s start countdown to end after the tone is over, or you can immediately press the start button to begin the game. The object of the game is to put all the interactable cubes in the scene back into the floating adsorption boxes (anchors) on the in-field panels by hand. When the game starts, the total time spent on the current level and the progress made in placing back the small cubes are displayed in the upper left corner. When all cubes have been moved and placed, the timer ends and the total time used is displayed. Currently, there are three levels of the object interactive game, each containing three and six and nine interactive cubes and adsorption anchor points, respectively. The in-game scene during gameplay is shown in Figure 11.

Under the Unity software environment (Unity 2022 LTS, Barracuda 3.0.0, Ultraleap Tracking SDK 6.14.0), performance metrics such as latency, inference time, or frame rate results (e.g., average inference time per frame, Unity FPS) are shown in Table 10. Under the specified software and hardware environment, this system meets the real-time interaction requirements in terms of model reasoning efficiency, frame rate, and latency (usually requiring latency < 100 ms and frame rate > 30 FPS), proving that it has excellent real-time performance.

The operational workflow of the system allows users to launch the main interface directly from the start page or access educational resources, including stroke rehabilitation videos and detailed FMA guides. Upon startup, users can navigate to specific modules, such as the FMA-based gesture assessment, the comprehensive algorithmic assessment, or the game interface. Within the game mode, users can select between gesture recognition and object interaction tasks. This automation of training and assessment processes creates a more conducive environment for patient rehabilitation.

## 7. Conclusions

By using Leap Motion’s non-contact sensing and clinical validation scales (BRS-H, FMA, ARAT), this study implements a ResNet-based gesture recognition module, combined with Unity to develop a new ResNet-based hand function assessment and rehabilitation training system for stroke patients. By using the ResNet gesture recognition model combined with the C# algorithm for gesture pose detection component for gesture recognition through double-listening verification, the accuracy and immediacy of the rehabilitation feedback is improved. A combination of quantitative assessment and rehabilitation training games is realized to promote patients’ engagement. The proposed system demonstrates the potential to meet the needs of stroke patients to rehabilitate in their home and community living environments by providing a more cost-effective and accessible assessment and assisted rehabilitation system. The developed system provides a validated technical framework for accurate movement pattern detection and assessment in real time. While current results verify the system’s feasibility under standard lighting conditions, future work will focus on conducting large-scale clinical trials with unseen stroke patients. Additionally, considering that vision-based sensors can be sensitive to external factors, future research will strictly analyze the system’s reliability in varying environments, specifically addressing performance in weak lighting and constrained spatial settings. Future work will also include a comprehensive comparison of computational efficiency and performance against other state-of-the-art architectures, such as transformers and graph-based approaches and comparative study with evaluations such as FMA and ARAT carried out by clinicians, to further enhance the system’s responsiveness. This will allow for a strictly subject-independent evaluation to further validate the system’s generalization capability across a broader range of pathological hand deformities and verify the therapeutic effectiveness.

Furthermore, acknowledging that challenges regarding device availability and technical support may arise in practical applications, future work will also dedicate efforts to gradually improving and verifying the system’s operability and establishing a supportive framework for home and community users.

## Figures and Tables

**Table 1:** Comparison of two approaches to functional hand rehabilitation.

Approaches	Advantages	Disadvantages
Rehabilitation based on gesture recognition and computer vision techniques	Non-invasive; no additional equipment to wear; easy to use	High external environmental requirements; low recognition speed and accuracy; tagging may be required
Wearable device-based hand function rehabilitation robot	More gestures can be recognized with small input data and high real-time accuracy; good robustness	More expensive; requires wearing additional equipment; prone to fatigue; requires calibration

**Table 2:** Limb movement characteristics of Brunnstrom’s stroke recovery stage (BRS-H).

Stages of Recovery	Limb Movement Characteristics	Hand Movement Characteristics
Flaccid Phase	No movement.	No movement.
Spasticity Phase	Joint reaction and joint movement.	Only slight flexion and extension.
Spastic Phase	Obvious convulsions.Limb movement can be controlled within a certain range through joint movement.	Hook grip could be performed, but fingers could not extend.
Late Spasticity Phase	Spasticity is weakened, limb movement can be carried out within a certain limit, and actions that are difficult to complete before being achieved.	The thumb can be pinched and released laterally, and the fingers can be extended semi-randomly in a small area, but they do not have the ability to grip.
Recovery Phase	Spasticity decreased, the patient can freely carry out collaborative movement, and can achieve independent joint movement and complex movement combination.	The five fingers of the patient can be flexed and extended at will, and spherical and cylindrical grips can be made, but the range of motion is limited.
Late Recovery Phase	The spasticity disappears and the patient has normal movement and coordination.	All grips can be completed, but the speed and accuracy have some gaps compared to the accuracy and speed of the limbs.

**Table 3:** Sequence to hand skeletal joint point mapping in LMDHG.

Line Segment (Sequence) Numbering			Mapped Hand Bone Joint Point Connections
Left hand	Palm core structure	1	Palm position → Wrist position
		2	Wrist position → Elbow position
		3	Wrist position → Thumb metacarpal
		4	Wrist position → Metacarpal of little finger
		5	Elbow position → Thumb metacarpal
		6	Elbow position → Metacarpal of little finger
	Thumb finger bone hierarchy connection	7	Thumb metacarpal→ Proximal phalanx of the thumb; this item is 0 for the thumb
		8	Proximal phalanx of the thumb → Middle phalanx of the thumb
		9	Middle phalanx of the thumb → Distal phalanx of the thumb
	Interphalangeal joint	10	Metacarpals of the thumb → Metacarpals of the index finger
	Index finger bone hierarchy connection	11	Metacarpal bones of the index finger → Proximal phalanx of the index finger
		12	Proximal phalanx of the index finger → Middle phalanx of the index finger
		13	Middle phalanx of the index finger → Distal phalanx of the index finger
	Interphalangeal joint	14	Metacarpals of the index finger → Metacarpals of the middle finger
	Middle finger bone hierarchy connection	15	Metacarpals of the middle finger → Proximal phalanx of the middle finger
		16	Proximal phalanx of the middle finger → Middle phalanx of the middle finger
		17	Middle phalanx of the middle finger → Distal phalanx of middle finger
	Interphalangeal joint	18	Metacarpals of the middle finger → Metacarpals of the ring finger
	Ring finger bone hierarchy connection	19	Metacarpals of the ring finger → Proximal phalanx of the ring finger
		20	Proximal phalanx of ring finger → Middle phalanx of ring finger
		21	Middle phalanx of ring finger → Distal phalanx of ring finger
	Interphalangeal joint	22	Metacarpal bone of ring finger → Metacarpal bone of little finger
	Pinky finger skeletal hierarchy connection	23	Metacarpals of the little finger → proximal phalanx of the little finger
		24	Proximal phalanx of little finger → middle phalanx of little finger
		25	Middle phalanx of little finger → Distal phalanx of little finger
Right hand	Detailed connections are consistent with the left hand	26–50	Detailed connections are the same as the left hand

**Table 4:** Selected experts from the FMA scale assessments addressed in this study.

**Wrist Sitting Position**		**None**	**Partial**	**Complete**
Alternate wrist flexion and extension	Inability to move at will	0	—	—
	Only partially performs full range of active wrist flexion and extension	—	1	—
	Fluently and adequately performs full range of active wrist flexion and extension	—	—	2
**Hand Sitting position**		**None**	**Partial**	**Complete**
Group flexion	No active flexion	0	—	—
	Can actively flex but not sufficiently	—	1	—
	Can actively flex sufficiently compared to the healthy side	—	—	2
Group extension	No active extension	0	—	—
	Can actively extend but not sufficiently or can relax actively flexed fingers	—	1	—
	Can actively extend sufficiently compared to the healthy side	—	—	2
Hook grip 2–5 interphalangeal joint flexion, metacarpophalangeal extension	Cannot maintain the required movement position	0	—	—
	Can maintain a hooked grip, but with weak grip strength	—	1	—
	Can maintain a hooked grip with a high degree of resistance	—	—	2
Lateral pinch (thumb pronation)	Cannot perform side pinching movements	0	—	—
	Can hold a piece of paper, but cannot resist pulling	—	1	—
	Can hold a piece of paper with resisting a large tensile force	—	—	2
Opposite pinch	Cannot perform a pair pinch movement	0	—	—
	Can hold a pencil, but cannot resist pulling	—	1	—
	Can hold a pencil with resisting tension	—	—	2
Column grip	Cannot perform a cylindrical grasp	0	—	—
	Can hold a cylindrical object, but cannot resist pulling	—	1	—
	Can hold a cylindrical object with resisting tension	—	—	2
Ball grip	Cannot perform a ballistic grasp	0	—	—
	Can hold a ballistic object, but cannot resist pulling	—	1	—
	Can hold a ballistic object with resisting tension	—	—	2

**Table 5:** Composition of hand skeletal joint point dataset.

Static Gesture Categories	Frame Number
Wrist Backward Extension	35,075
Wrist Forward Extension	29,814
Hand Group Flexion	39,955
Hand Group Extension	17,420
Hooked Grip	55,897
Side Squeeze (Thumb Inward)	12,335
Pair Squeeze	39,042
Index Finger Pointing	15,300
Pointing (Thumb Up)	6191
Pointing (Thumb Down)	18,367
OK Gesture	87,288
Palm Open Up	58,223
Palm Open Down	11,129
Fist Clenching	27,764
Rocking Gesture	48,601

**Table 6:** Ablation experimental programme and result.

Programme	Basic MLP	1D-CNN	LSTM	Residual Block Connectivity	Focal Loss	Macro F1	Val Acc
1	√	×	×	×	×	78.3%	74.6%
2	√	√	×	×	×	86.4%	84.2%
3	√	√	×	√	×	89.1%	87.3%
4	√	√	×	×	√	89.4%	88.2%
5	√	√	×	√	√	91.0%	90.9%
6	√	√	√	×	×	90.5%	90.3%

**Note**: “√” denotes the presence of the corresponding component, whereas “×” denotes its absence.

**Table 7:** Normal range of motion values for key hand and upper limb joints.

Joint/Motion Part/Description	Motion Direction	Normal Joint Range of Motion Value/Description
Shoulder	Bend forward (lift your arms forward to the top of your head)	180°
	Stretch back (arms stretch back)	60°
	Abduction (arms raised sideways to the top of the head)	180°
	Internal rotation (back of hand against waist, elbow forward)	70°
Elbow	External rotation (palm up, elbow pushed back)	90°
	Buckling (hand touching shoulder)	150°
Forearm	Stretch (fully extended)	0°
	Forward rotation (palm down)	90°
Wrist	Supination (palms up)	90°
	Palm flexion (palms facing arms)	80°
	Extend your back (the back of your hand is bent into your arms)	70°
	Radial deviation (hand leaning to thumb side)	20°
Fingers	Foot deviation (hand leaning to the little finger side) Metacarpophalangeal flexion (clenched fist)	30°
	Flexion of metacarpophalangeal joint (clenching fist)	90°
	Metacarpophalangeal joint extension (finger extension)	30°
	Proximal interphalangeal joint flexion	100°
Thumb	Distal interphalangeal joint flexion	90°
	Carpometacarpal joint abduction (thumb away from palm)	70°
	Flexion of metacarpophalangeal joint	50°
	Interdigital joint flexion	80°
	Palm opposition function (the tip of thumb can touch the root of little finger)	
Special Functional References	Palm arch state maintenance	Palm arch state maintenance

**Table 8:** Key clinical evaluation criteria.

Assessment Instrument	Core Requirements
FMA	Shoulder–elbow coordinated flexion (0–150°), dorsal extension of wrist ≥ 70°, finger clenched fist ≥ 90°
ARAT	Thumb to palm can touch the root of little finger, flexion of metacarpophalangeal joint/proximal interphalangeal joint of finger ≥ 90°
BRS-H (Stages V–VI)	(Stages V–VI) dorsal extension of wrist ≥ 60°, distal interphalangeal joint/proximal interphalangeal joint separation movement of wrist and finger
MMT (Muscle Strength ≥ 3)	Full range anti-gravity motion (e.g., 150° elbow flexion, 70° dorsal extension of wrist)

**Table 9:** Functional thresholds (based on ARAT/FMA).

Assessment Items	Thresholds/Criteria	Clinical Significance
Dorsal extension of wrist	≥60°	ARAT grasping and releasing function is up to standard
Flexion of metacarpophalangeal joints of fingers	≥80°	Minimum angle required for daily activities such as holding a cup
Thumb to palm	Can touch the metacarpophalangeal joint of the little finger	Fine operation ability (BRS-H ≥ stage IV)

**Table 10:** System real-time performance metrics.

Performance Metric	Test Condition	Average (Range)
Model Inference Time	GPU Inference (Barracuda, RTX 4060)	1.8 ms (1.0–2.5 ms)
	CPU Inference (ONNX Runtime, i9-14900HX)	3.2 ms (2.5–4.5 ms)
Unity Engine Frame Rate	FMA Module Scene	61 FPS (55–70 FPS)
	Rehabilitation Game Module Scene	58 FPS (52–65 FPS)
End-to-End System Latency	Sensor to Algorithm Output Latency	13.3 ms (11–18 ms)
Total Perceived Latency (Motion to Screen Feedback)		27 ms (23–31 ms)

## Data Availability

Part of the data used in this study are publicly available from the IRISA-KIHT-S dataset [36]. The remaining data were collected by the authors and are available from the corresponding author upon reasonable request. These data are not publicly available due to data privacy considerations.
